# Risk Factors for E-Cigarette, or Vaping, Product Use–Associated Lung Injury (EVALI) Among Adults Who Use E-Cigarette, or Vaping, Products — Illinois, July–October 2019

**DOI:** 10.15585/mmwr.mm6845e1

**Published:** 2019-11-15

**Authors:** Livia Navon, Christopher M. Jones, Isaac Ghinai, Brian A. King, Peter A. Briss, Karen A. Hacker, Jennifer E. Layden

**Affiliations:** ^1^Illinois Department of Public Health; ^2^Center for Preparedness and Response, CDC,^3^National Center for Injury Prevention and Control, CDC; ^4^Epidemic Intelligence Service, CDC; ^5^National Center for Chronic Disease Prevention and Health Promotion, CDC.

The United States is experiencing an unprecedented outbreak of e-cigarette, or vaping, product use–associated lung injury (EVALI) (*1*). All EVALI patients have used e-cigarette, or vaping, products, and most (≥85%) have reported using products containing tetrahydrocannabinol (THC) (*2*,*3*), the principal psychoactive component of cannabis. To examine whether e-cigarette, or vaping, product use behaviors differed between adult EVALI patients and adults who use these products but have not developed lung injury, the Illinois Department of Public Health (IDPH) conducted an online public survey during September–October 2019 targeting e-cigarette, or vaping, product users in Illinois. Among 4,631 survey respondents, 94% reported using any nicotine-containing e-cigarette, or vaping, products in the past 3 months; 21% used any THC-containing products; and 11% used both THC-containing products and nicotine-containing products. Prevalence of THC-containing product use was highest among survey respondents aged 18–24 years (36%) and decreased with increasing age. E-cigarette, or vaping, product use behaviors of 66 EVALI patients aged 18–44 years who were interviewed as part of the ongoing outbreak investigation were compared with a subset of 519 survey respondents aged 18–44 years who reported use of THC-containing e-cigarette, or vaping, products. Compared with these survey respondents, EVALI patients had higher odds of reporting exclusive use of THC-containing products (adjusted odds ratio [aOR] = 2.0, 95% confidence interval [CI] = 1.1–3.6); frequent use (more than five times per day) of these products (aOR = 3.1, 95% CI = 1.6–6.0), and obtaining these products from informal sources, such as a dealer, off the street, or from a friend (aOR = 9.2, 95% CI = 2.2–39.4). The odds of using Dank Vapes, a class of largely counterfeit THC-containing products, was also higher among EVALI patients (aOR = 8.5, 95% CI = 3.8–19.0). These findings reinforce current recommendations not to use e-cigarette, or vaping, products that contain THC and not to use any e-cigarette, or vaping, products obtained from informal sources. In addition, because the specific compound or ingredient causing lung injury is not yet known, CDC continues to recommend that persons consider refraining from use of all e-cigarette, or vaping, products while the outbreak investigation continues (*1*).

IDPH developed an online public survey targeting Illinois adults who use e-cigarette, or vaping, products based on the structured questionnaire developed by IDPH and administered to EVALI patients as part of the ongoing outbreak investigation. The public survey included questions about the types of e-cigarette, or vaping, products survey respondents used in the past 3 months, where these products were obtained, combustible cigarette and marijuana use, and any reported illness associated with e-cigarette, or vaping, product use. The public survey link was posted on the IDPH website during September 17–October 8, 2019 and was publicized through the media, posted on IDPH social media accounts, and promoted by local health departments (*4*). Because of an IDPH Institutional Review Board determination, the survey was restricted to persons aged ≥18 years.

To compare survey respondents with EVALI patients, a subset of respondents with similar characteristics to those of EVALI patients was selected. Data were available for 137 EVALI patients reported to IDPH; 15% (20 of 137) were aged <18 years; of adult EVALI patients, 97% (113 of 117) were aged 18–44 years (Supplementary Figure, https://stacks.cdc.gov/view/cdc/82320).* Among EVALI patients aged 18–44 years, 66 of 113 (58%) had the structured patient questionnaire administered either via telephone, by a public health staff member (53 of 66, 80%); during an in-person interview, usually by a health care provider (nine of 66, 14%); or online (four of 66; 6%) (*3*). Among these 66 EVALI patients, 85% reported use of THC-containing e-cigarette, or vaping, products. Based on these characteristics of EVALI patients (i.e. primarily adults aged <44 years with high THC-containing product use prevalence), survey respondents for the comparative analysis were limited to those aged 18–44 years who reported use of THC-containing e-cigarette, or vaping, products. Survey respondents were further restricted to those who resided in one of the 28 Illinois counties with any reported EVALI cases and who did not report seeking health care for illness compatible with EVALI. All interviewed EVALI adult patients aged 18–44 years were included in the comparative analysis.

Survey results were summarized with descriptive statistics. P-values were assessed using Pearson’s chi-square test; for cells with small numbers, Fisher’s exact test was used. To compare EVALI patients with the subset of survey respondents that reported using THC-containing products, aORs were calculated using multivariable logistic regression models that controlled for race/ethnicity and age group. P-values <0.05 were considered statistically significant. Analyses were conducted using SAS (version 9.4; SAS Institute).

Among 7,704 survey respondents, 4,631 (60%) met the study inclusion criteria (i.e., Illinois residents aged ≥18 years who completed demographic questions, reported use of e-cigarette, or vaping, products in the past 3 months, and did not have EVALI) (Supplementary Figure, https://stacks.cdc.gov/view/cdc/82320).^†^ The median age of included respondents was 38 years (range = 18–83 years), 3,035 (66%) were men, and 3,932 (89%) identified as non-Hispanic white (white) (Table 1). Overall, 3,641 (94%) respondents reported using any nicotine-containing e-cigarette, or vaping, products in the preceding 3 months, including 3,222 (84%) who reported exclusive use of nicotine-containing products. Use of any THC-containing e-cigarette, or vaping, products was reported by 930 (21%) of survey respondents, including 212 (5%) who used such products exclusively. Use of both nicotine-containing and THC-containing products was reported by 418 (11%) survey respondents. Prevalence of THC-containing product use decreased with increasing age: 36% and 13% of respondents aged 18–24 years and ≥45 years, respectively, reported using THC-containing products. Use of nicotine-containing products was consistent across age groups (93%–96%). Among survey respondents, use of combustible marijuana (24%) was higher than that of combustible tobacco (7%).

**TABLE 1:** E-cigarette, or vaping, and combustible product use among survey respondents aged ≥18 years who used e-cigarettes during the 3 months preceding the survey (N = 4,631), by age group, sex, and race/ethnicity — Illinois, July–October 2019*

Characteristic	No./Total no. (%)							
	E-cigarette, or vaping, product use					Combustible product use		All respondents
	THC-containing only^†^	Nicotine-containing only^†^	Both THC- and nicotine-containing^†^	Any nicotine-containing	Any THC-containing	Marijuana	Cigarettes	
**Age group (yrs)**								
18–24	29/443 (7)	306/443 (69)	108/443 (24)	414/443 (93)	206/571 (36)	264/592 (45)	56/592 (9)	**601 (13)**
25–34	72/1,036 (7)	845/1,036 (82)	119/1,036 (11)	964/1,036 (93)	289/1,236 (23)	353/1,256 (28)	83/1,256 (7)	**1,273 (27)**
35–44	54/1,238 (4)	1,053/1,238 (85)	131/1,238 (11)	1,185/1,239 (96)	264/1,422 (19)	309/1,437 (21)	77/1,437 (5)	**1,457 (31)**
≥45	57/1,135 (5)	1,018/1,135 (90)	60/1,135 (5)	1,078/1,135 (95)	171/1,283 (13)	193/1,291 (15)	93/1,290 (7)	**1,300 (28)**
**Sex**								
Men	119/2,530 (5)	2,118/2,530 (84)	293/2,530 (12)	2,412/2,531 (95)	603/2,959 (20)	740/3,002 (25)	163/3,002 (5)	**3,035 (66)**
Women	93/1,322 (7)	1,104/1,322 (84)	125/1,322 (9)	1,229/1,322 (93)	327/1,553 (21)	379/1,574 (24)	146/1,573 (9)	**1,596 (34)**
**Race/Ethnicity^§,^** ^¶^								
White	165/3,304 (5)	2,789/3,304 (84)	350/3,304 (11)	3,140/3,305 (95)	757/3,836 (20)	919/3,885 (24)	252/3,884 (6)	**3,932 (89)**
Black	6/60 (10)	42/60 (70)	12/60 (20)	54/60 (90)	24/74 (32)	26/78 (33)	10/78 (13)	**79 (2)**
Other	12/149 (8)	119/149 (80)	18/149 (12)	137/149 (92)	47/183 (26)	57/187 (30)	13/187 (7)	**188 (4)**
Hispanic	22/181 (12)	135/181 (75)	24/181 (13)	159/181 (88)	63/215 (29)	67/219 (31)	18/219 (8)	**221 (5)**
**All respondents**	**212/3,852 (5)**	**3,222/3,852 (84)**	**418/3,852 (11)**	**3,641/3,853 (94)**	**930/4,512 (21)**	**1,119/4,576 (24)**	**309/4,575 (7)**	**4,631**

**Abbreviation:** THC = tetrahydrocannabinol.

* Online survey responses were collected during September 17–October 8, 2019.

^†^ Only survey respondents who answered both the question about use of THC-containing e-cigarette products (n = 4,512) and the question about nicotine-containing e-cigarette products (n = 3,853) were used to calculate these mutually exclusive categories.

^§^ Whites, blacks, and persons of other races were non-Hispanic; Hispanic persons could be of any race.

^¶^ Race/ethnicity data was missing for 211 survey respondents.

Approximately 82% of male survey respondents aged 18–34 years reported frequent (more than five times per day) use of nicotine-containing e-cigarette, or vaping, products, compared with 76% of women of the same age (Table 2). Among adults aged 18–34 years, the prevalence of frequent use of THC-containing e-cigarette, or vaping, products was twice as high among men (25%) as among women (13%). Among survey respondents who reported any use of THC-containing products, exclusive use was reported by a higher proportion of women than of men both among those aged 18–34 years (26% versus 17%) and among those aged ≥35 years (31% versus 22%). A similar proportion of male and female survey respondents aged 18–34 years obtained THC-containing products from informal sources (a dealer, friends, or on the street) (72% and 68%, respectively); however, among adults aged ≥35 years, men were more likely to report informal sources of THC-containing products (56%) than were women (39%).

**TABLE 2:** E-cigarette, or vaping, product use behaviors among survey respondents aged ≥18 years who used e-cigarettes during the 3 months preceding the survey (N = 4,631), by age group and sex — Illinois, July–October 2019*

E-cigarette, or vaping, use behavior	18–34 years (n = 1,874)			≥35 years (n = 2,757)			All ages		
	No./Total no. (%)		P-value^†^	No./Total no. (%)		P-value^†^	No./Total no. (%)		P-value^†^
	Men (n = 1,283)	Women (n = 591)		Men (n = 1,752)	Women (n = 1,005)		Men (n = 3,035)	Women (n = 1,596)	
**Any nicotine-containing products**	964/1,020 (95)	414/459 (90)	0.002	1,448/1,511 (96)	815/863 (94)	0.12	2,412/2,531 (95)	1,229/1,322 (93)	0.003
Only nicotine-containing products	809/964 (84)	342/414 (83)	0.55	1,309/1,448 (90)	762/815 (94)	0.01	2,118/2,412 (88)	1,104/1,229 (90)	0.07
Any nicotine-containing product <1x/day^§^	21/956 (2)	19/407(5)	0.01	17/1,428 (1)	10/800 (1)	0.90	36/2,382 (2)	24/1,202 (2)	0.28
Any nicotine-containing product >5x/day^§^	780/956 (82)	309/407 (76)	0.02	1,271/1,428 (89)	663/800 (83)	<0.0001	2,051/2,384 (86)	972/1,207 (82)	<0.0001
**Any THC-containing products**	321/1,243 (26)	174/564 (31)	0.03	282/1,716 (16)	153/989 (15)	0.51	603/2,959 (20)	327/1,553 (21)	0.59
Only THC-containing products	56/321 (17)	45/174 (26)	0.03	63/282 (22)	48/153 (31)	0.04	119/603 (20)	93/327 (28)	0.003
Any THC-containing product <1x/day^§^	64/255 (25)	44/123 (36)	0.03	74/220 (34)	36/110 (33)	0.87	138/475 (29)	80/233 (34)	0.15
Any THC-containing product >5x/day^§^	64/255 (25)	16/123 (13)	0.007	40/220 (18)	24/110 (22)	0.43	104/475 (22)	40/233 (17)	0.14
Dank Vapes^¶^	102/240 (42)	51/126 (40)	0.71	53/223 (24)	19/105 (18)	0.25	155/463 (33)	70/231 (30)	0.40
Obtained any THC-containing product informally**	172/240 (72)	82/120 (68)	0.51	118/210 (56)	42/107 (39)	0.004	290/450 (64)	124/227 (55)	0.01
**Both THC- and nicotine-containing products**	155/1,020 (15)	72/459 (16)	0.81	138/1,510 (9)	53/863 (6)	0.01	293/2,530 (12)	125/1,322 (9)	0.04

**Abbreviations:** CI = confidence interval; THC = tetrahydrocannabinol.

* Online survey responses were collected during September 17–October 8, 2019.

^†^ Calculated using Pearson’s chi-square test.

^§^ Frequency of use was reported by individual product. If any e-cigarette, or vaping, product was reported as being used more than five times a day, the survey respondent was classified as using that class of product (nicotine- or THC-containing) more than five times/day. The same criteria were used to classify product use as less than one time/day.

^¶^ Dank Vapes are a class of largely counterfeit THC-containing products of unknown provenance that are marketed under a common name and distributed through informal sources.

** Obtaining any THC-containing e-cigarette, or vaping, products from informal sources (a dealer, off the street, or from a friend) was compared with obtaining any THC-containing products from a formal source (store or licensed dispensary). Because online sources might be formal (e.g., a licensed dispensary) or informal, persons who reported online purchases were excluded from this analysis. Fewer than 1% of public survey respondents reported online purchases.

Among the 4,631 survey respondents, 519 (11%) met the additional age, THC-use, and county of residence criteria for the comparative analysis with the 66 interviewed EVALI patients aged 18–44 years. Significant demographic differences between EVALI patients and this subset of survey respondents were identified (Table 3). Compared with the subset of survey respondents, EVALI patients had higher odds of being aged <30 years (odds ratio [OR] = 6.0, 95% CI = 3.1–11.5) and of identifying as a racial/ethnic group other than white (OR = 2.9, 95% CI = 1.7–5.2). Among EVALI patients who used THC-containing e-cigarette, or vaping, products, the odds for frequent use of these products were significantly higher compared with the subset of THC-using survey respondents (aOR = 3.1, 95% CI = 1.6–6.0). In addition, the odds were significantly higher among EVALI patients for exclusive use of THC-containing e-cigarette, or vaping, products (aOR = 2.0, 95% CI = 1.1–3.6) and obtaining THC-containing products through informal sources versus from a licensed dispensary or store^§^ (aOR = 9.2, 95% CI = 2.2–39.4). Compared with the subset of survey respondents, EVALI patients also had higher odds of reporting use of Dank Vapes (aOR = 8.5, 95% CI = 3.8–19.0), a class of largely counterfeit THC-containing products of unknown provenance that are marketed under a common name and distributed through informal sources (*5*).

**TABLE 3:** Characteristics of e-cigarette, or vaping, product use behaviors among adult* EVALI patients and survey respondents^†^^,^^§^ who reported using tetrahydrocannabinol (THC)-containing products — Illinois, July–October 2019

Characteristic	No./Total no. (%)		Odds ratio (95% CI)^¶^	P-value^¶^	Adjusted odds ratio (95% CI)**	P-value
	EVALI patients (n = 66)	Survey respondents (n = 519)				
**Sex**						
Men	49/66 (74)	341/519 (66)	1.6 (0.8–2.7)	0.17	1.6 (0.9–3.0)	0.11
Women	17/66 (26)	178/519 (34)	reference	—^††^	—^††^	—^††^
**Age group (yrs)**						
18–29	54/66 (82)	222/519 (43)	6.0 (3.1–11.5)	<0.0001	—**	—**
30–44	12/66 (18)	297/519 (57)	reference	—^††^	—^††^	—^††^
**Race/Ethnicity**						
All other racial/ethnic groups^§§^	23/66 (35)	87/519 (17)	2.9 (1.7–5.2)	0.0001	—**	—**
Unknown	6/66 (9)	22/519 (4)	3.0 (1.2–7.9)	0.03	—**	—**
White, non-Hispanic	37/66 (56)	410/519 (79)	reference	—^††^	—^††^	—^††^
**E-cigarette, or vaping, use behavior**						
**Any nicotine-containing products**	45/66 (68)	237/361 (66)	1.1 (0.6–2.0)	0.69	1.1 (0.6–1.9)	0.87
Only nicotine-containing products	10/45 (22)	0/237 (0)	—^¶¶^	—^¶¶^	—^¶¶^	—^¶¶^
Any nicotine-containing product <1x/day^,^***^,†††^	5/42 (12)	16/232 (7)	1.8 (0.5–5.6)	0.34	1.4 (0.5–4.2)	0.57
Any nicotine-containing product >5x/day***	27/42 (64)	178/232 (77)	0.5 (0.3–1.1)	0.09	0.8 (0.4–1.7)	0.57
**Any THC-containing products** ^¶¶^	56/66 (85)	519/519 (100)	—^¶¶^	—^¶¶^	—^¶¶^	—^¶¶^
Only THC-containing products	21/56 (38)	124/519 (24)	1.9 (1.1–3.4)	0.03	2.0 (1.1–3.6)	0.03
Any THC-containing product <1x/day***	7/49 (14)	122/403 (30)	0.4 (0.2–0.9)	0.02	0.4 (0.2–1.0)	0.04
Any THC-containing product >5x/day***	19/49 (39)	76/403 (19)	2.7 (1.5–5.1)	0.001	3.1 (1.6–6.0)	0.0009
Dank Vapes^§§§^	45/53 (85)	140/391 (36)	10.1 (4.6–22.0)	<0.0001	8.5 (3.8–19.0)	<0.0001
Obtained any THC-containing product informally^¶¶¶^	48/50 (96)	251/378 (66)	12.1 (2.9–50.8)	<0.0001	9.2 (2.2–39.4)	0.003
**Both THC- and nicotine-containing products**	35/66 (53)	237/361 (66)	0.59 (0.3–1.0)	0.05	0.56 (0.3–1.0)	0.05

**Abbreviations:** CI = confidence interval; EVALI = E-cigarette, or vaping, product use–associated lung injury; THC = tetrahydrocannabinol.

* Online survey responses were collected during September 17–October 8, 2019. Survey respondents were asked about e-cigarette, or vaping, product use in the 3 months preceding survey completion; EVALI patients were asked about e-cigarette, or vaping, product use in the 3 months preceding symptom onset.

^†^ Aged 18–44 years.

^§^ Only survey respondents who resided in one of the 28 Illinois counties with any reported outbreak-associated EVALI cases during July 31-October 15, 2019 were included in this analysis.

^¶^ Calculated using Pearson’s chi-square test.

** Adjusted for race/ethnicity and age group. Each adjusted odds ratio used the age group ≥30 years and non-Hispanic white as the reference group. Therefore, adjusted odd ratios for age groups and race/ethnicity are not presented.

^††^ Values were not calculated for reference cells.

^§§^ Includes survey respondents who identified as Hispanic, non-Hispanic black, and non-Hispanic other.

^¶¶^ Only survey respondents who reported using THC-containing e-cigarette, or vaping, products in the past 3 months were included in this analysis, therefore, odds ratios were not calculated for this e-cigarette, or vaping, use behavior.

*** Frequency of use was reported by individual product. If any e-cigarette, or vaping, product was reported as being used more than five times a day, the survey respondent or case were classified as using that class of product (e.g., nicotine- or THC-containing) more than five times/day. The same criteria were used to classify product use frequency as less than one time/day).

^†††^ Because of small cell size, Fisher’s exact test was used to calculate the 95% CI and p-value for the unadjusted odds ratio.

^§§§^ Dank Vapes are a class of largely counterfeit THC-containing products of unknown provenance that are marketed under a common name and distributed through informal sources.

^¶¶¶^ Obtaining any THC-containing e-cigarette, or vaping, products from informal sources (a dealer, off the street, or from a friend) was compared with obtaining any THC-containing products from a formal source (store or licensed dispensary). Because online sources might be formal (e.g., a licensed dispensary) or informal, persons who reported online purchases were excluded from this analysis. No EVALI patients and <1% of public survey respondents reported online purchases.

## Discussion

Since the introduction of e-cigarettes into the United States in 2007, use of these devices has increased rapidly, particularly among youths (*6*). Although initially created for use with nicotine-containing products, e-cigarettes are also used to aerosolize THC (*7*). In this survey of Illinois residents who used e-cigarette, or vaping, products and did not have EVALI, use of THC-containing products was less prevalent (21%) than was use of nicotine-containing products (94%); however, a higher proportion of survey respondents aged <35 years reported using THC-containing products, consistent with the observed age distribution of EVALI patients in this outbreak both in Illinois and nationally (*1*,*2*). Two thirds of survey respondents were men, reflecting the sex distribution of outbreak-associated EVALI patients, in Illinois and nationally (*1*,*2*). Among persons aged 18–34 years, the prevalence of frequent daily use of both nicotine-containing and THC-containing e-cigarette, or vaping, products was higher among men than among women. These findings suggest that e-cigarette, or vaping, product use behaviors among younger adults, especially men, might place them at higher risk for developing EVALI associated with this outbreak.

A much higher proportion of adult EVALI patients reported use of THC-containing e-cigarette, or vaping, products (85%) than did adults who use e-cigarette, or vaping, products and have not developed lung injury (21%). When e-cigarette, or vaping, product use among EVALI patients aged 18–44 years was compared with that of a subset of survey respondents aged 18–44 years who reported use of THC-containing products, a number of significant differences were found. Specifically, patients with EVALI had higher odds of reporting exclusive use of THC-containing products, as well as reporting frequent use of these products, obtaining them through informal sources, and using a counterfeit THC-containing product marketed as Dank Vapes. Because the comparative analysis was restricted to survey respondents who reported using THC-containing e-cigarette, or vaping products, the calculated ORs comparing THC-containing product use behaviors between EVALI patients and survey respondents are likely conservative. 

The findings in this report are subject to at least six limitations. First, the survey was restricted to persons aged ≥18 years and findings might not be representative of younger persons; 15% of EVALI patients in Illinois during July–October 2019 were aged <18 years. Second, survey respondents were self-selected and might not be representative of the overall population of persons who use e-cigarette, or vaping, products in Illinois. To address this potential for bias, the comparative analysis was restricted to survey respondents in the same age group, geographic areas of residence, and with similar types of product use as those of EVALI patients and was adjusted for higher survey response rates among whites and older adults. Third, only 58% of Illinois EVALI patients aged 18–44 years have been interviewed; this nonresponse rate might introduce selection bias, although the characteristics of interviewed patients were similar to those of all reported EVALI patients. Fourth, EVALI patients who reported exclusive use of nicotine-containing products were also included in the comparative analysis with the subset of survey respondents who reported use of THC-containing products. Including these EVALI patients might have introduced bias, however, the prevalence of using nicotine-containing products was similar among the two groups. In addition, because analysis of product use behaviors was limited to only those persons who reported using a specific product (e.g., THC product use behaviors were only compared among EVALI patients and survey respondents who reported using THC-containing products) the inclusion of these EVALI patients did not affect the analysis of THC-containing product use behaviors. Fifth, although a similar survey instrument was used with EVALI patients and online survey respondents, most EVALI patients were interviewed by public health staff members via telephone. Differences in data collection methodology might have affected reporting of product use behaviors by EVALI patients compared with that of anonymous online survey respondents. Finally, these data were only collected from Illinois residents. Illinois has a comprehensive medical marijuana program in place but has not yet implemented sales of marijuana for recreational use; the legal purchase of tobacco products is restricted to persons aged ≥21 years. E-cigarette, or vaping, product use behaviors likely vary by jurisdictional policies that control access to these products; this might limit the generalizability of the results in this report. 

This is the first report to analyze e-cigarette, or vaping, product use behaviors associated with increased risk of EVALI during this outbreak. The use of an anonymous public survey facilitated the rapid collection of data to inform the ongoing investigation. Differences were observed in e-cigarette, or vaping, product use behaviors between adults who use THC-containing e-cigarette, or vaping, products and patients with EVALI. The findings in this report reinforce current recommendations that persons should not use e-cigarette, or vaping, products that contain THC, or any e-cigarette, or vaping, products obtained from informal sources such as off the street, from a dealer, or from a friend. In addition, because the specific compound or ingredient causing lung injury is not yet known, CDC continues to recommend that persons consider refraining from use of all e-cigarette, or vaping, products while the outbreak investigation continues (*1*).

**Summary**

**What is already known about this topic?**

Most U.S. patients with e-cigarette, or vaping, product use–associated lung injury (EVALI) report using tetrahydrocannabinol (THC)-containing e-cigarette, or vaping, products. Product use behaviors that increase risk for EVALI are unknown.

**What is added by this report?**

Compared with survey respondents aged 18–44 years reporting using of THC-containing e-cigarette, or vaping, products, EVALI patients aged 18–44 years had higher odds of reporting exclusive and frequent use of THC-containing products and obtaining these products from informal sources, such as a dealer, off the street, or from a friend, and of using Dank Vapes, a class of largely counterfeit THC-containing products.

**What are the implications for public health practice?**

CDC recommends not using THC-containing e-cigarette, or vaping, products, or any e-cigarette, or vaping, products obtained from informal sources.

## Footnotes

* EVALI cases were reported to the Illinois Department of Public Health during July 31–October 15, 2019, from 28 counties. These counties accounted for an estimated 83% of the Illinois population in 2018.

† Thirty-two survey respondents were aged <18 years; 1,800 resided in states other than Illinois or did not confirm Illinois residency, and age or gender information was missing for 1,120 respondents. Respondents who reported no e-cigarette, or vaping, product use in the past 3 months (n = 106) or who visited an emergency department and/or were hospitalized for vaping-related symptoms (n = 15) were excluded.

§ No EVALI patients in Illinois have reported purchasing THC-containing e-cigarette, or vaping, products online. Among public survey respondents who reported using THC-containing e-cigarette, or vaping, products, five of 519 (1%) reported online purchase of dry marijuana herb, butane hash oil, or THC-containing prefilled cartridges. Online sites likely represent a mix of illicit and licit sources; therefore, respondents who purchased THC-containing products online were not included in the comparison of informal to formal place of e-cigarette, or vaping, product purchase.
